# Prevalence of spine degeneration diagnosis by type, age, gender, and obesity using Medicare data

**DOI:** 10.1038/s41598-021-84724-6

**Published:** 2021-03-08

**Authors:** Chantal S. Parenteau, Edmund C. Lau, Ian C. Campbell, Amy Courtney

**Affiliations:** Exponent, Inc., 3350 Peachtree Road NE, Suite 1125, Atlanta, GA 30326 USA

**Keywords:** Epidemiology, Skeleton, Disability, Medical imaging, Epidemiology, Population screening, Body mass index, Obesity

## Abstract

Identifying the prevalence of degenerative spinal pathologies and relevant demographic risk factors is important for understanding spine injury risk, prevention, treatment, and outcome, and for distinguishing acute injuries from degenerative pathologies. Prevalence data in the literature are often based on small-scale studies focused on a single type of pathology. This study evaluates the prevalence of diagnosis of selected degenerative spinal pathology diagnoses using Medicare insurance claim data in the context of published smaller-scale studies. In addition, the data are used to evaluate whether the prevalence is affected by age, sex, diagnosed obesity, and the use of medical imaging. The Medicare Claims 5% Limited Data Set was queried to identify diagnoses of degenerative spinal pathologies. Unique patient diagnoses per year were further evaluated as a function of age, gender, and obesity diagnosis. Participants were also stratified by coding for radiological imaging accompanying each diagnosis. The overall prevalence of diagnosed spinal degenerative disease was 27.3% and increased with age. The prevalence of diagnosed disc disease was 2.7 times greater in those with radiology. The results demonstrate that degenerative findings in the spine are common, and, since asymptomatic individuals may not receive a diagnosis of degenerative conditions, this analysis likely underestimates the general prevalence of these conditions.

## Introduction

The spine is the central supporting structure of the torso; it routes and protects the spinal cord while providing for flexibility and shock absorption. In evaluating spine trauma, it is relevant to understand the prevalence of degenerative spine pathologies. Spine degeneration affects both the mechanical properties and anatomic morphology of the vertebrae, discs, and surrounding soft tissues. Such changes can affect the range of motion, loading patterns, and tolerance to traumatic events involving loads substantially greater than those applied during common activities. The sequelae of some degenerative pathologies may be misidentified as acute injuries if the underlying process was not previously identified.

Types of spinal degenerative pathologies examined in this study include stenosis, spine curvatures (kyphosis, lordosis, and scoliosis), diffuse idiopathic skeletal hyperostosis (DISH), spondylitis, osteoporosis, and disc degeneration. The prevalence of these pathologies was further evaluated based on age, sex, and obesity. With more than a third of the total US population aged 65 or older^1^, and with the high prevalence of obesity^2^, it is relevant to identify any association between these objective demographic factors and the prevalence of spine degeneration. Additionally, it was also relevant to investigate whether radiology influences the rate of diagnosis of conditions because of the possibility of incidental findings.

Stenosis refers to narrowing of the spinal canal or neural foramina. Stenosis can be congenital but is commonly degenerative in origin. It may be asymptomatic but is often associated with numbness or tingling.

Kyphosis, lordosis, and scoliosis are types of spine curvature. Kyphosis generally refers to a convex curvature of the spine in the sagittal plane; a diagnosis typically refers to excessive kyphosis of the upper thoracic spine. Scoliosis is curvature of the thoracic or lumbar spine in the coronal plane, whether toward the left (levoscoliosis) or the right (dextroscoliosis). A diagnosis of lordosis typically refers to excessive concave curvature of the lumbar spine in the sagittal plane.

Diffuse idiopathic skeletal hyperostosis (DISH) is a disorder characterized by excess bone formation and continuous ossification of soft tissue structures along the anterior and lateral aspects of the spine, generally with preservation of intervertebral disc height and apophyseal joints^3^. Other terms used to identify DISH include ankylosing hyperostosis, Forestier disease, and Forestier-Rotes-Querol disease^4,5^. DISH occurs most commonly in the thoracolumbar spine. Ossification of the posterior longitudinal ligament (OPLL) is often linked to DISH, more commonly in the cervical region.

Spondylitis refers to inflammation of the joints of the spine. Inflammation may result when ligaments, tendons, or joints of the spine are acutely injured. Inflammation of the spine may be associated with systemic inflammatory diseases. One type of spondylitis with biomechanical significance is ankylosing spondylitis (AS), also known as Bechterew’s disease, which can cause inflammation in the sacroiliac joints and in vertebrae and spinal ligaments.

Osteoporosis results from a chronic imbalance between rates of bone resorption and bone deposition. A decrease in bone mineral density of trabecular bone, such as in a vertebral body, results in disproportionately larger reduction in load tolerance^6^.

Degeneration of intervertebral discs is a common finding in the spine^7,8^ and may include pathologies such as disc bulges, disc herniations, osteophyte formation, loss of disc height, and disc desiccation. Osteophytes, or bone spurs, often protrude from the edges of vertebral endplates and sometimes bridge the intervertebral disc space. Osteophytes develop in response to alterations in localized pressure.

Medicare insurance claims represent a large database that can provide insight into the prevalence of spine degeneration and associated demographic factors. The objective of this study is to assess the prevalence of specific diagnoses pertaining to spinal degeneration as a function of age, sex, and obesity, as well as an investigation of the influence of radiological imaging on the rate of diagnosis of such conditions. The results are discussed in comparison to published research pertaining to the prevalence of specific diagnoses.

## Results

The Medicare Claims 5% Limited Data Set (LDS) represents about 1.6–1.8 million individuals per calendar year. It included 21,771,202 person-years, with 12,162,068 female and 9,609,134 male person-years, where a person-year is defined as a single Medicare enrollee per calendar year. Table 1 shows the total number of enrollee-years by sex and spine degeneration diagnosis and prevalence by age group. More than one-third of cumulative enrollees were in the 65–69 age group, irrespective of sex. The most prevalent diagnosis overall was disc disease, and DISH was least common. Online Appendices 2–9 provide additional data.

**Table 1:** Cumulative number and frequency of enrollees in person-years by spine degeneration type, age group, and sex.

	All	Females	Males	All	Females	Males
**Age groups**	**Total person-years**					
n	21,771,202	12,162,068	9,609,134			
**Freq.** 65–69	37.2%	34.4%	40.7%			
70–74	20.9%	20.0%	22.1%			
75–79	16.3%	16.3%	16.2%			
80–84	12.5%	13.4%	11.4%			
85+	13.1%	15.9%	9.7%			
	**w/any spine degeneration**			**w/stenosis**		
n	5,956,687	4,223,190	1,744,872	992,521	601,050	391,471
65–69	27.0%	26.0%	29.5%	26.0%	24.9%	27.7%
70–74	22.5%	21.9%	24.0%	23.0%	22.3%	24.0%
75–79	20.0%	19.9%	20.2%	20.9%	20.8%	21.1%
80–84	16.0%	16.5%	14.9%	16.5%	17.1%	15.7%
85+	14.5%	15.8%	11.4%	13.6%	15.0%	11.5%
	**w/spine curvature**			**w/DISH**		
n	25,813	18,802	7,011	3,896	1,607	2,289
65–69	25.3%	23.8%	29.1%	32.2%	33.7%	31.2%
70–74	21.7%	21.3%	22.8%	24.0%	22.9%	24.8%
75–79	19.6%	19.6%	19.8%	20.4%	19.5%	20.9%
80–84	16.1%	16.6%	14.7%	14.0%	14.8%	13.4%
85 +	17.4%	18.7%	13.7%	9.4%	9.1%	9.7%
	**w/spondylitis**			**w/disc degeneration**		
n	39,478	21,694	17,784	2,653,433	1,636,634	1,016,799
65–69	33.9%	33.1%	34.9%	30.3%	29.3%	32.0%
70–74	24.4%	23.7%	25.2%	23.6%	22.9%	24.6%
75–79	18.5%	18.4%	18.6%	19.5%	19.4%	19.7%
80–84	12.8%	13.1%	12.5%	14.6%	15.1%	13.8%
85+	10.4%	11.7%	8.7%	12.0%	13.3%	10.0%
	**w/osteoporosis**			**w/other**		
n	1,935,767	1,731,451	204,316	320,217	219,379	100,838
65–69	22.5%	22.8%	20.2%	29.2%	28.8%	30.2%
70–74	20.5%	20.5%	20.7%	24.1%	23.5%	25.4%
75–79	20.2%	20.1%	21.2%	20.1%	20.0%	20.3%
80–84	18.0%	17.8%	19.1%	14.6%	14.8%	14.1%
85+	18.8%	18.8%	18.7%	12.0%	12.9%	10.1%

**Figure 1:**
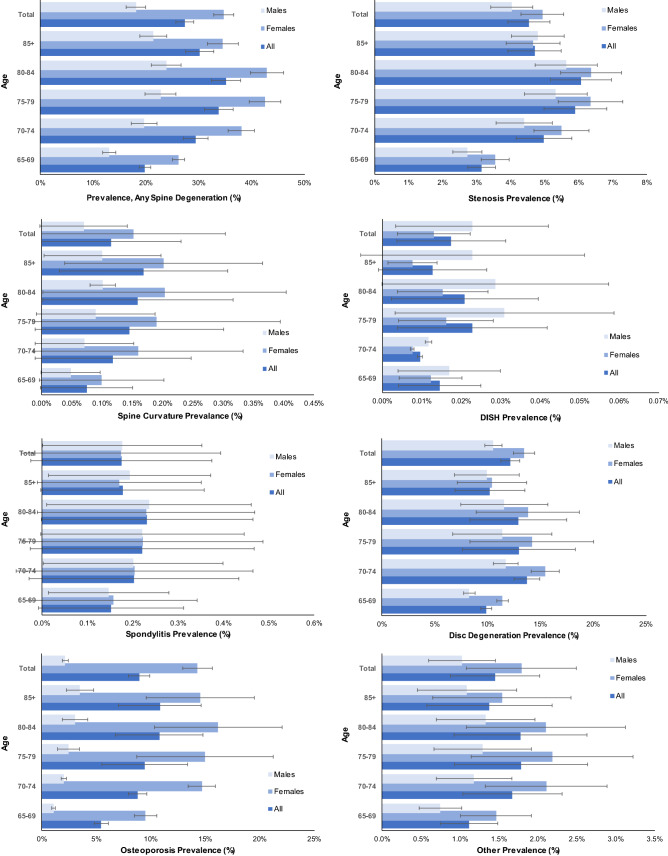
Spine degeneration diagnosis prevalence by age group and sex.

Figure 1 shows the prevalence of spine degeneration diagnoses by age group, sex, and type. The yearly prevalence of spine degeneration was 27.3 ± 1.7% overall (mean ± standard deviation among years). It was greater in females than in males at 34.7 ± 1.9% versus 18.1 ± 1.8%. Overall, the prevalence was 4.5 ± 0.6% for stenosis, 0.11 ± 0.12% for spine curvature, 0.017 ± 0.014% for DISH, 0.17 ± 0.20% for spondylitis, 12.2 ± 0.9% for disc degeneration, 8.9 ± 1.0% for osteoporosis, and 1.4 ± 0.6% for other spine degeneration.

**Figure 2:**
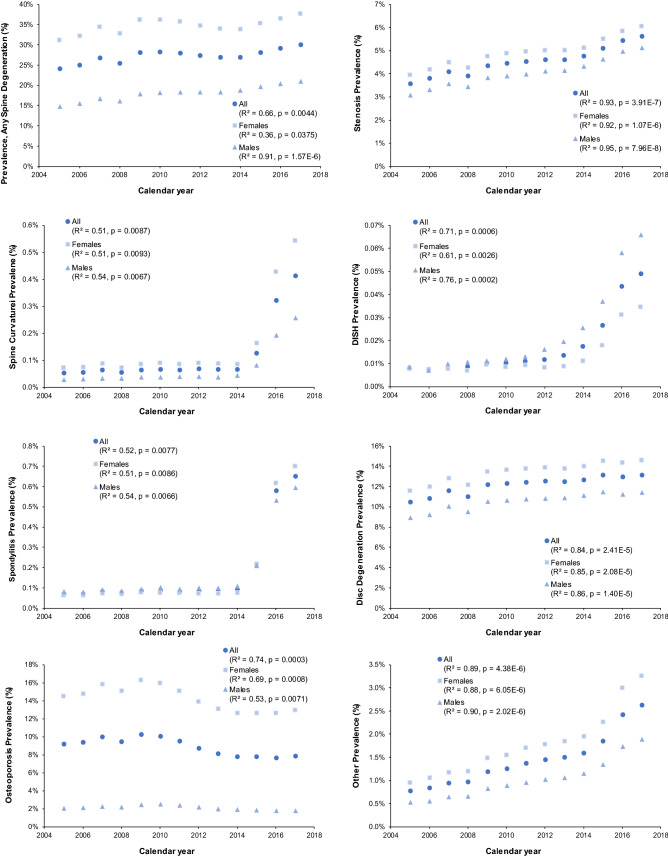
Spine degeneration diagnosis prevalence by type, calendar year, and sex. Pearson’s Correlation coefficients and *p* values are indicated.

Figure 2 shows the prevalence of degenerative spine diagnoses by calendar year, type, and sex. In general, the prevalence of each pathology increased with calendar year. Overall, the prevalence increased from 24.2% in 2005 to 30.1% in 2017. There was negligible increase observed for osteoporosis or disc degeneration. The trends in increasing prevalence by year were similar for males and females. The prevalence of all diagnoses evaluated were consistently higher for females than for males except for DISH.

**Figure 3:**
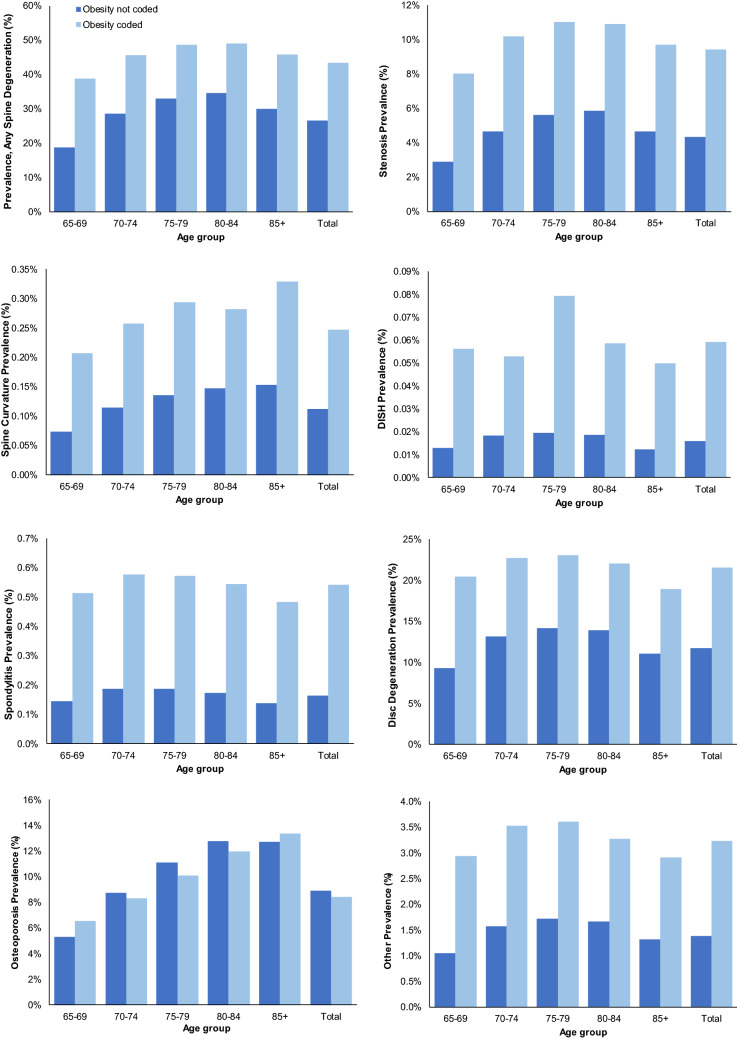
Spine degeneration diagnosis prevalence by type, age, and obesity coding.

Figure 3 shows the prevalence of spine degeneration type for enrollees coded as obese and those not coded as obese. The prevalence of spine disease was greater with obesity for all spine disease types, except osteoporosis. The increase was greatest for DISH, irrespective of age group. Overall, the prevalence ratio of degenerative spine pathologies for obese versus non-obese enrollees was 2.19 for stenosis, 2.21 for spine curvature, 3.75 for DISH, 3.32 for spondylitis, 1.84 for disc degeneration, 0.94 for osteoporosis, and 2.34 for other spine pathologies. Online Appendix 10 reports the number of enrollees by spine degeneration type, age group, sex, and obesity coding.

**Figure 4:**
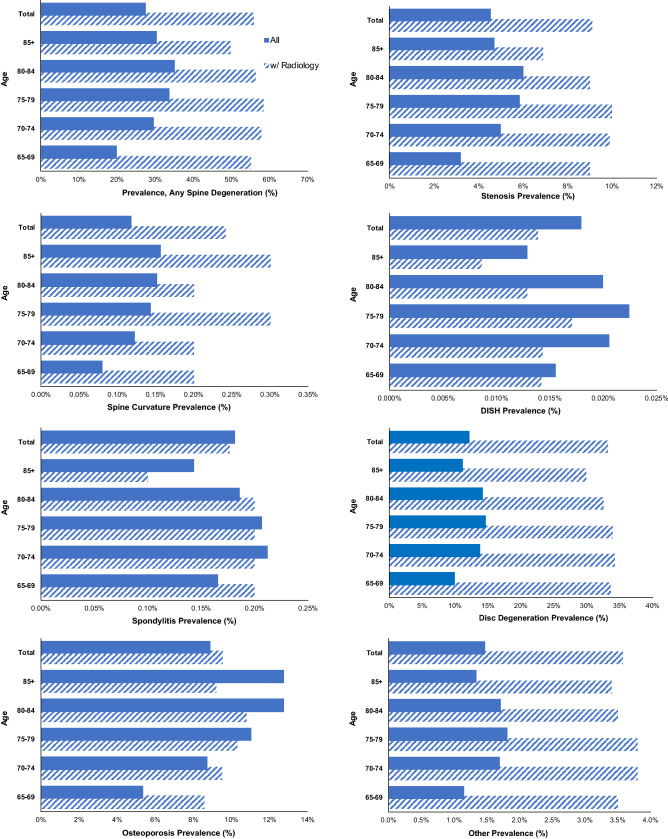
Prevalence of degenerative spine diagnoses by type and age in overall sample and in radiology subsample.

Figure 4 compares the prevalence of degenerative spine diagnoses in the overall sample and in the sub-sample with radiology. Online Appendix 11 shows the number of enrollees with a history of spine radiology broken out by spine degeneration type and age group. The prevalence of diagnosed disc disease was 2.7× greater in those with radiology. The prevalence of diagnosed spondylitis and osteoporosis were similar in the overall group compared to the subgroup with radiology.

## Discussion

In this study, diagnostic codes were used to identify the prevalence of degenerative spinal pathologies and differences based on age, sex, and obesity from Medicare data. A strength of this analysis is the large volume of data. More than 20 million person-years were evaluated; more than 8 million had diagnoses of spine degeneration. Prior studies generally investigated much smaller groups and focused on one spinal pathology, sometimes in the context of risk factors or common comorbidities. Additionally, most prior studies rely on data collected at a single institution, and depending on the local population could be skewed by factors such as demographics, economics, or predominant types of occupations. The Medicare Claims 5% LDS is advantageous not just because of its absolute size, but also because it represents the elderly U.S. population from all geographic areas and the full range of socioeconomic backgrounds. Online Appendix 12 provides a more extensive summary of literature data.

Various demographic and comorbid factors are associated with spinal degeneration^9–11^. By definition, degenerative changes develop over time; increased prevalence of degenerative spine pathology with age is therefore expected and is well documented^7,8,12^. Increasing age has been specifically associated with increased spine curvature^13–15^, stenosis^15–17^, endplate sclerosis, disc degeneration^7^, osteophyte formation, DISH, AS, and reduced bone mineral density^17,18^.

With the exception of DISH, the analysis consistently indicates a higher prevalence of degenerative spine diagnoses in females compared to males. One possible factor is the higher prevalence of osteoporosis in older females compared to males. Looker et al. (2017) reported that 11.6% of females and 3.6% of males age 50+ had osteoporosis of the lumbar spine^19^. The gap between sexes age 65+ was even larger in the Medicare data. Middle-aged and older women are more susceptible to osteoporosis than men and younger women because of a perimenopausal increase in the rate of bone resorption.

In the present study, the prevalence of diagnosed spine degeneration was higher in obese individuals; some of the increased prevalence over time may be associated with increased prevalence of obesity in the United States^2^. It is likely that some enrollees who were obese were not coded as such, which would have reduced the strength of the observed association between obesity and the respective degenerative spinal diagnosis.

The prevalence of degenerative spine diagnoses was generally higher in individuals who underwent spine imaging procedures compared to those who did not. While no restriction was placed on the medical reason for ordering spine imaging, these patients do not represent a random sample of those over 65 because radiology was ordered for some reason. It is notable that spine imaging is not part of routine annual health checks.

In this study, we only analyzed individuals aged 65 and older, the age at which most U.S. legal residents become eligible for coverage. While the nuances of eligibility for Medicare and how coverage works for individuals with additional private health insurance are beyond the scope of this work, broadly speaking, the vast majority individuals over this age in the U.S. will have their medical claims submitted to Medicare. The Medicare program is almost universal among elderly citizens in the U.S. It provides both inpatient and outpatient services and is nationwide in geographic coverage. Thus, healthcare service claims derived from the Medicare system broadly represents a wide spectrum of the US elderly population. Healthcare service and diagnosis derived from Medicare data can therefore correctly reflect the prevalence of conditions, including spine pathologies, among this population. Although some individuals below the age of 65 are also covered by Medicare, we have excluded them from this study because the reasons that one might qualify for Medicare below the age of 65 typically involve receiving Social Security Disability Insurance, which would bias the population of these individuals.

The Medicare data suggest a very low prevalence of 0.017 ± 0.014% for DISH overall. The prevalence was 0.008% in 2005 and 0.049% in 2017, possibly reflecting increased use of medical imaging and/or recognition of the pathology. Prevalences reported in studies focused on DISH are much higher and vary from 4% to as high as 35% in individuals older than 70^4,20–24^. Whatever the reason for the discrepancy, there are likely many times more individuals in the population with undiagnosed DISH than with diagnoses.

The prevalence was highest for disc degeneration (12.2 ± 0.9%). These values are lower than those reported in the literature (Online Appendix 12). For example, Boden et al. (1990) indicated that 80% of asymptomatic patients 60 years and older had cervical disc degeneration^25^. In our study, those who had radiological imaging had considerably higher prevalence of diagnosed degenerative disc disease (33.2% vs 12.2% overall).

The prevalence was 4.5 ± 0.6% for spinal stenosis and increased to 9.1% in the sub-sample with imaging (10.9% in females and 13.6% in males). In the literature, prevalence of stenosis is often reported in the cervical and lumbar spine (Online Appendix 12). Shim et al. (2009) reported a prevalence of 4% to 9% in the cervical spine based on cadaveric data^26^. Wang et al. (2019) reported a prevalence in individuals age 74 and older of 38.6%^27^. Yabuki et al. (2013) estimated a prevalence of 5.7% for stenosis in the lumbar spine^28^. The prevalence of acquired stenosis in the lumbar spine has been reported to increase with age and body mass index^29,30^. Kalichman et al. (2009) reported that lumbar stenosis prevalence was similar for asymptomatic and symptomatic individuals^29^.

The prevalence of spine degeneration was second highest for osteoporosis (8.9 ± 1.0%). The prevalence of osteoporosis was higher in females than males (14.3% vs. 2.1%). Looker et al. (2017) reported that 8% of adults over 50 had osteoporosis^19^. This is consistent with our overall analysis and may be somewhat lower because of the inclusion of people between age 50 and 65. They also reported a higher prevalence in females (11.6%) compared to males (3.6%). Other studies have reported higher prevalences for certain demographics (Online Appendix 12).

The prevalence of degenerative spine diagnoses also increased over time from 24.2% in 2005 to 30.1% in 2017 and was greatest for DISH. There was no increase observed for osteoporosis or disc degeneration. Increased use of medical imaging may help explain the increased prevalence of degenerative spine diagnoses over time. Imaging studies allow the identification of spine degeneration in asymptomatic individuals as well as incidental findings in individuals with unrelated complaints. Since radiology is a primary diagnostic tool for most degenerative spinal pathologies, these are likely underreported in the Medicare database for those without spine imaging. Ishimoto et al. (2012) compared the prevalence of lumbar stenosis using imaging and symptoms-related data collected during a series of physical activities^31^. Results indicated that prevalence was 8.23× greater in the imaging sample than in the self-reported sample (76.5% vs. 9.3%).

A marked increase in the prevalence of diagnosis of spine curvature, DISH, and spondylitis is observed starting around 2015 and subsequently continuing to increase year upon year. While not certain, it seems unlikely that the true prevalence of these degenerative conditions suddenly increased. Instead, the data may be explained by increased awareness of spinal degenerative pathologies, increased use of diagnostic radiology, increased prevalence of obesity, the increasing specificity of ICD-10 coding, or even possibly changes in reimbursement procedures. Of note, individuals with radiological imaging were actually less likely to be diagnosed with DISH than those without, so increased use of diagnostic radiology cannot explain this phenomenon. Similarly, although the switch from ICD-9 to ICD-10 coding occurred around the time of this increase, diagnoses of DISH continue to increase year-on-year after 2015, suggesting that this phenomenon is not purely an artifact of the new diagnostic coding.

Differences between literature and Medicare data may be related to the criteria used to identify spine degeneration. For example, stenosis is often assessed using the Torg-Pavlov ratio^32–34^. Spine curvature is quantified using metrics like the Cobb angle. DISH is generally diagnosed using Resnick’s criteria (Online Appendix 12)^3^. We cannot verify that these criteria were consistently applied for all diagnoses in the Medicare database.

Osteoporosis is well-documented to result in reduced load tolerance of affected bones. Vertebral ossification stiffens the spine and may redistribute loadbearing to other regions. Spine curvature results in asymmetric loading of vertebrae that can accelerate degenerative changes due to repetitive loading^35^. Degeneration of intervertebral discs or spine curvature also alters the pressure distribution on the associated vertebrae and stimulates the formation of osteophytes^36,37^.

Associations between spine degeneration and traumatic spine injury have been documented. For example, Viano et al. (2019) and Davis et al. (2019) reported that the presence of spinal degeneration was associated with spinal fracture-dislocation and spinal cord injury in rear-impact motor vehicle crashes with characteristics such that these injuries were unexpected^38,39^. Spine ossification can result in serious sequelae, including quadriplegia and atlantoaxial subluxation^40,41^. Stenosis can increase the risk of neurological injuries in traumatic events^42^. Studies indicate that the risk of vertebral fractures is also associated with stenosis^43–45^, kyphosis^13,46^, and DISH^38,39,47^. Fused segments resulting from DISH, AS, or other ossification can become brittle and more prone to fracture in relatively minor trauma^48^.

Yoganandan et al. (1989) tested spines with and without spine degeneration and reported lower biomechanical tolerance with degeneration^49^. A complication of decreased load tolerance in the spine is increased prevalence of vertebral fractures. Vertebral fracture has been reported to be the most common fragility fracture, yet it is often unrecognized and therefore underdiagnosed^50^. The risk of vertebral fractures is associated with a number of degenerative diagnoses, especially osteoporosis (Online Appendix 12).

The Medicare data and literature reviewed in the present study indicate that degenerative spine pathology is common, and that prevalence increases with age and obesity. The results indicate that degenerative spine pathologies may not be diagnosed prior to medical imaging studies being performed. The first medical evaluation to identify a degenerative spine disorder may come after a traumatic event that results in a fracture when one might not otherwise be expected. For less prevalent diagnoses like DISH and AS, the increased risk of fracture should inform medical providers evaluating patients with vertebral fracture resulting from relatively low trauma.

## Materials and methods

The Medicare Claims 5% limited data set (LDS) was queried on a yearly basis to identify enrollees with selected spine pathologies using ICD-9 and ICD-10 codes. The data set includes Medicare fee-for-service beneficiaries enrolled from January 1, 2005 to December 31, 2017. The LDS comprises a 5% random national sample of Medicare enrollees and provides their associated physician service claims. Individuals younger than 65 years of age were excluded.

The prevalence of specific diagnoses were calculated based upon ICD-9 and ICD-10 codes corresponding to each of seven categories of degenerative spine pathologies. SAS Software version 9.4 was used to query the sample. The total number of enrollees included in the 5% LDS was determined for each year, as well as each individual’s age and sex. The number diagnosed with degenerative spinal pathologies was quantified, categorized as: stenosis; spine curvature; DISH; spondylitis; disc degeneration; osteoporosis; and other pathologies. ICD-9 codes were used from 2005 to the end of September 2015, and ICD-10 codes were used for from October 1, 2015 and onward. The specific ICD-9 or ICD-10 codes are presented in Online Appendix 1, Table A1-1. Trends in diagnoses over time were evaluated using Pearson’s correlation coefficient.

Regardless of the number of medical records for a particular individual, each enrollee in the 5% LDS sample was summarized by the presence or absence of a diagnosis each calendar year. Medical billing codes were used to identify concurrent spine degeneration and obesity diagnoses each year. Both primary and secondary diagnoses were considered to identify each condition. The 5% LDS typically follows the same individuals for multiple years until their death. Because the 5% LDS is provided on an annual basis and because individual enrollees may be included in multiple years, data were summarized as “person-years.” Prevalence of each spine degeneration diagnosis was determined by dividing the number of enrollees diagnosed with that disorder by the total number of enrollees in the sample.

For sub-group analysis, data was divided into the following age groups: 65–69, 70–74, 75–79, 80–84 and 85+ years old. The data was also grouped by enrollees who had concurrent coding for obesity (Table A1-1). A subsample of individuals with spine degeneration and who received spine radiology (X-ray, CT scans, or MR imaging) was also identified (Table A1-2). There was no restriction on the medical reason for the imaging being ordered. The prevalence of spine degeneration in those with both recorded diagnoses and spine imaging was compared to the overall results.

## Supplementary Information

Supplementary Information

## Data Availability

The Medicare 5% LDS is available from the U.S. Government at https://www.cms.gov/Research-Statistics-Data-and-Systems/Files-for-Order/LimitedDataSets, and all relevant analyses are summarized in tables in the Online Appendices.

## References

[1] Cheng JS, Forbes J, Wong C, Perry E, Wang M, Lu Y, Anderson D, Mummaneni P (2014). The epidemiology of adult spinal deformity and the aging population. Minimally Invasive Spinal Deformity Surgery.

[2] Ogden, C. L., Carroll, M. D., Fryar, C. D. & Flegal, K. M. Prevalence of obesity among adults and youth: United States, 2011–2014. U.S. Department of Health and Human Services, NCHS Data Brief No. 219. https://www.cdc.gov/nchs/products/databriefs/db219.htm (2015). 26633046

[3] Resnick D, Niwayama G (1976). Radiographic and pathologic features of spinal involvement in diffuse idiopathic skeletal hyperostosis (DISH). Radiology.

[4] Boachie-Adjei O, Bullough PG (1987). Incidence of ankylosing hyperostosis of the spine (Forestier’s disease) at autopsy. Spine.

[5] Resnick D, Shaul SR, Robins JM (1975). Diffuse idiopathic skeletal hyperostosis (DISH): Forestier’s Disease with extraspinal manifestations. Radiology.

[6] Myers ER, Wilson SE (1997). Biomechanics of osteoporosis and vertebral fracture. Spine.

[7] Brinjikji W (2015). Systematic literature review of imaging features of spinal degeneration in asymptomatic populations. AJNR Am. J. Neuroradiol..

[8] Theodore N (2020). Degenerative cervical spondylosis. NEJM.

[9] Nachemson A, Schultz AB, Berkson MH (1979). Mechanical properties of human lumbar spine motion segments: Influences of age, sex, disc level, and degeneration. Spine.

[10] Greaves LL (2009). Pediatric and adult three-dimensional cervical spine kinematics: Effect of age and sex through overall motion. Spine.

[11] Wong HK, Hui JH, Rajan U, Chia HP (2005). Idiopathic scoliosis in Singapore schoolchildren: A prevalence study 15 years into the screening program. Spine.

[12] Benoist M (2003). Natural history of the aging spine. Eur. Spine J..

[13] Ailon T, Shaffrey CI, Lenke LG, Harrop JS, Smith JS (2015). Progressive spinal kyphosis in the aging population. Neurosurgery.

[14] Holcombe SA, Wang S, Grotberg JB (2017). Age-related changes in thoracic skeletal geometry of elderly females. Traffic Inj. Prev..

[15] Parenteau, C., Caird, M., Kohoyda-Inglis, C., Holcombe, S. & Wang, S. Characterization of thoracic spinal development by age and sex with a focus on occupant safety. SAE Technical Paper 2020-01-0520. 10.4271/2020-01-0520 (2020).

[16] Milne JS, Williamson J (1983). A longitudinal study of kyphosis in older people. Age Ageing.

[17] Parenteau, C. S., Holcombe, S., Zhang, P., Kokoyda-Inglis, C. & Wang, S. The effect of age on fat and bone properties along the vertebral spine. SAE Technical Paper 2013-01-1244. 10.4271/2013-01-1244 (2013).

[18] Agnusdei D (1998). Age-related decline of bone mass and intestinal calcium absorption in normal males. Calc Tiss Int..

[19] Looker AC, Isfahani NS, Fan B, Shepherd JA (2017). Trends in osteoporosis and low bone mass in older US adults, 2005–2006 through 2013–2014. Osteoporos Int..

[20] Cassim B, Mody GM, Rubin DL (1990). The prevalence of diffuse idoiopathic skeletal hyperostosis in African blacks. Rheumatology.

[21] Weinfeld R, Olson P, Maki D, Griffiths HJ (1997). The prevalence of diffuse idiopathic skeletal hyperostosis (DISH) in two large American Midwest metropolitan hospital populations. Skelet. Radiol..

[22] Kiss CS, O'Neill TW, Mituszova M, Szilágyi M, Poór G (2002). The prevalence of diffuse idiopathic skeletal hyperostosis in a population-based study in Hungary. Scand. J. Rheumatol..

[23] Kim SI, Ha KY, Lee JW, Kim YH (2018). Prevalence and related clinical factors of thoracic ossification of the ligamentum flavum—A computed tomography-based cross-sectional study. Spine J..

[24] Uehara M (2020). Prevalence of diffuse idiopathic skeletal hyperostosis in the general elderly population: A Japanese cohort survey randomly sampled from a basic resident registry. Clin. Spine Surg..

[25] Boden SD (1990). Abnormal magnetic-resonance scans of the cervical spine in asymptomatic subjects. A prospective investigation. J. Bone Jt. Surg. Am..

[26] Shim JH (2009). A comparison of angled sagittal MRI and conventional MRI in the diagnosis of herniated disc and stenosis in the cervical foramen. Eur. Spine J..

[27] Wang MC, Kreuter W, Wolfla CE, Maiman DJ, Deyo RA (2009). Trends and variations in cervical spine surgery in the United States: Medicare beneficiaries, 1992 to 2005. Spine.

[28] Yabuki S (2013). Prevalence of lumbar spinal stenosis, using the diagnostic support tool, and correlated factors in Japan: A population-based study. J. Orthop Sci..

[29] Kalichman L, Guermazi A, Li L, Hunter DJ (2009). Association between age, sex, BMI and CT-evaluated spinal degeneration features. J. Back Musculoskelet. Rehabil..

[30] Kalichman L, Kim DH, Li L, Guermazi A, Hunter DJ (2010). Computed tomography-evaluated features of spinal degeneration: prevalence, intercorrelation, and association with self-reported low back pain. Spine J..

[31] Ishimoto Y (2012). Prevalence of symptomatic lumbar spinal stenosis and its association with physical performance in a population-based cohort in Japan: The Wakayama Spine Study. Osteoarthr. Cartil..

[32] Torg JS (1986). Neurapraxia of the cervical spinal cord with transient quadriplegia. J. Bone Jt. Surg. Am..

[33] Torg JS (1996). The relationship of developmental narrowing of the cervical spinal canal to reversible and irreversible injury of the cervical spinal cord in football players. J. Bone Jt. Surg. Am..

[34] Pavlov H, Torg JS, Robie B, Jahre C (1987). Cervical spinal stenosis: Determination with vertebral body ratio method. Radiology.

[35] Bertram H (2006). Accelerated intervertebral disc degeneration in scoliosis versus physiological ageing develops against a background of enhanced anabolic gene expression. Biochem. Biophys. Res. Commun..

[36] Kumaresan S, Yoganandan N, Pintar FA, Maiman DJ, Goel VK (2001). Contribution of disc degeneration to osteophyte formation in the cervical spine: A biomechanical investigation. J. Orthop. Res..

[37] Nathan H (1962). Osteophytes of the vertebral column: an anatomical study of their development according to age, race, and sex with considerations as to their etiology and significance. J. Bone Jt. Surg..

[38] Viano, D., Parenteau, C. & White, S. Influence of DISH, ankylosis, spondylosis and osteophytes on serious-to-fatal spinal fractures and cord injury in rear impacts. SAE Technical Paper 2019-01-1028. 10.4271/2019-01-1028 (2019).

[39] Davis, M. S., Isaacs, J. L., Graber, M. A. & Fisher, J. L. Thoracic spine extension injuries in occupants with pre-existing conditions during rear end collisions. SAE Technical Paper 2019-01-1222. 10.4271/2019-01-1222 (2019).

[40] Pouchot J, Watts CS, Esdaile JM, Hill RO (1987). Sudden quadriplegia complicating ossification of the posterior longitudinal ligament and diffuse idiopathic skeletal hyperostosis. Arthr. Rheum..

[41] Oostveen JC, Van de Laar MA, Tuynman FH (1996). Anterior atlantoaxial subluxation in a patient with diffuse idiopathic skeletal hyperostosis. J. Rheumatol..

[42] Takao T (2013). Clinical relationship between cervical spinal canal stenosis and traumatic cervical spinal cord injury without major fracture or dislocation. Eur. Spine J..

[43] Bey T (1998). Spinal cord injury with a narrow spinal canal: Utilizing Torg’s Ratio method of analyzing cervical spine radiographs. J. Emerg. Med..

[44] Bailes JE (2005). Experience with cervical stenosis and temporary paralysis in athletes. J. Neurosurg. Spine.

[45] Zhang L (2012). Cervical spinal canal narrowing and cervical neurological injuries. Chin. J. Traumatol..

[46] Bruno AG, Anderson DE, D'Agostino J, Bouxsein ML (2012). The effect of thoracic kyphosis and sagittal plane alignment on vertebral compressive loading. J. Bone Miner. Res..

[47] Poncelet AN, Rose-Innes AP, Aminoff M, Josephson SA (2014). Neurologic disorders associated with bone and joint disease. Aminoff's Neurology and General Medicine.

[48] Hendrix RW, Melany M, Miller F, Rogers LF (1994). Fracture of the spine in patients with ankylosis due to diffuse skeletal hyperostosis: Clinical and imaging findings. Am. J. Roentgen.

[49] Yoganandan N, Ray G, Pintar FA, Myklebust J, Sances A (1989). Stiffness and strain energy criteria to evaluate the threshold of injury to an intervertebral joint. J. Biomech..

[50] Li Y (2018). The prevalence and under-diagnosis of vertebral fractures on chest radiograph. BMC Musculoskelet. Disord..

