# Supplementary material for: Prevalence of spine degeneration diagnosis by type, age, gender, and obesity using Medicare data
Source: Sci Rep. 2021 Mar 8;11:5389. doi: 10.1038/s41598-021-84724-6 (PMC7940625; doi:10.1038/s41598-021-84724-6)
Supplement: Supplementary file 1 — Supplementary Information [file 41598_2021_84724_MOESM1_ESM.docx]

**Prevalence of Spine Degeneration by Type, Age, Gender, and Obesity Using Medicare Data**

Chantal S. Parenteau, Ph.D., Edmund C. Lau, M.S., Ian C. Campbell, Ph.D., Amy Courtney, Ph.D.

**Appendix 1:** The following ICD-9 and ICD-10 diagnostic codes were used to identify individuals with spine degeneration, and the following CPT codes were used to identify individuals who had received radiological imaging. An individual was included in a disorder group a maximum of one time each year, even if a diagnostic code appeared in multiple medical records that year.

Table A1-1: ICD-9 and ICD-10 diagnostic codes used to identify spine degenerative pathologies.

| **Degenerative** | **Coding** | | **Description** |
| --- | --- | --- | --- |
| **Pathologies** | ICD-9 | ICD-10 |  |
| Stenosis | 723.0 |  | Spinal stenosis in cervical region |
|  | 724.0 |  | Spinal stenosis, other than cervical |
|  |  | M48.0 | Spinal stenosis |
| Spinal Curvature | 737.19 |  | Other kyphosis (acquired) |
|  | 737.29 |  | Other lordosis (acquired) |
|  | 737.39 |  | Other kyphoscoliosis and scoliosis |
|  | 737.40 |  | Curvature of spine, unspecified, associated with other conditions |
|  | 737.41 |  | Kyphosis associated with other conditions |
|  | 737.42 |  | Lordosis associated with other conditions |
|  | 737.8 |  | Other curvatures of spine |
|  | 737.9 |  | Unspecified curvature of spine |
|  |  | M40.29 | Other kyphosis |
|  |  | M40.5 | Lordosis, unspecified |
|  |  | M41.8 | Other forms of scoliosis |
|  |  | M43.9 | Deforming dorsopathy, unspecified |
| DISH | 721.6 |  | Ankylosing vertebral hyperostosis |
|  | 723.7 |  | Ossification of posterior longitudinal ligament in cervical region |
|  |  | M48.1 | Ankylosing hyperostosis [Forestier] |
|  |  | M48.8x2 | Other specified spondylopathies, cervical region |
|  |  | M67.88 | Other specified disorders of synovium and tendon, other side |
| Spondylitis | 720.0 |  | Ankylosing spondylitis |
|  | 720.9 |  | Spondylitis |
|  |  | M45 | Ankylosing spondylitis |
|  |  | M46.8 | Other specified inflammatory spondylopathies |
|  |  | M46.9 | Unspecified inflammatory spondylopathy |
| Disc Degeneration | 721.0 |  | Cervical spondylosis without myelopathy |
|  | 721.1 |  | Cervical spondylosis with myelopathy |
|  | 721.2 |  | Thoracic spondylosis without myelopathy |
|  | 721.3 |  | Lumbosacral spondylosis without myelopathy |
|  | 721.4 |  | Thoracic or lumbar spondylosis with myelopathy |
|  | 721.8 |  | Spondylosis - other allied disorders |
|  | 721.9 |  | Spondylosis of unspecified site |
|  | 722.0 |  | Displacement of cervical intervertebral disc without myelopathy |
|  | 722.1 |  | Displacement of thoracic or lumbar intervertebral disc without myelopathy |
|  | 722.3 |  | Schmorl's nodes |
|  | 722.4 |  | Degeneration of cervical intervertebral disc |
|  | 722.5 |  | Degeneration of thoracic or lumbar intervertebral disc |
|  | 722.6 |  | Degeneration of intervertebral disc, site unspecified |
|  | 722.7 |  | Intervertebral disc disorder with myelopathy |
|  | 722.9 |  | Other and unspecified disc disorder |
|  |  | M47 | Spondylosis |
|  |  | M50 | Cervical disc disorders |
|  |  | M51 | Thoracic, thoracolumbar, and lumbosacral intervertebral disc disorders |
| Osteoporosis | 733.0 |  | Osteoporosis |
|  |  | M80 | Osteoporosis with current pathological fracture |
|  |  | M81 | Osteoporosis without current pathological fracture |
| Other | 720.1 |  | Spinal enthesopathy |
|  | 720.8 |  | Other inflammatory spondylopathies |
|  | 723.8 |  | Other syndromes affecting cervical region |
|  | 723.9 |  | Unspecified osteochondropathy |
|  | 738.4 |  | Acquired spondylolisthesis |
|  | 721.5 |  | Kissing spine |
|  |  | M42.1 | Adult osteochondritis of spine |
|  |  | M42.9 | Spinal osteochondritis, unspecified |
|  |  | M43.1 | Spondylolisthesis |
|  |  | M46.0 | Spinal enthesopathy |
|  |  | M48.2 | Kissing spine |
|  |  | M48.4 | Fatigue fracture of vertebra |
|  |  | M48.5 | Collapsed vertebra, not elsewhere classified |
|  |  | M49.8 | Spondylopathies in diseases classified elsewhere |
| Obesity | 278.00 |  | Obesity, unspecified |
|  | 278.01 |  | Morbid obesity |
|  | 278.03 |  | Obesity hypoventilation syndrome |
|  | V85.3 |  | Body mass index between 30-39, adult |
|  | V85.4 |  | Body mass index 40 and over, adult |
|  |  | E66.0 | Obesity due to excess calories |
|  |  | E66.1 | Drug-induced obesity |
|  |  | E66.2 | Morbid (severe) obesity with alveolar hypoventilation |
|  |  | E66.8 | Other obesity |
|  |  | E66.9 | Obesity, unspecified |
|  |  | Z68.3 | Body mass index (BMI) 30-39, adult |
|  |  | Z68.4 | Body mass index (BMI) 40 or greater, adult |

Table A1-2: CPT codes used to identify radiological imaging of the spine.

| CPT Code | Definition |
| --- | --- |
| 72010 | Radiologic examination, spine, entire, survey study |
| 72020 | Radiologic examination, spine, single view, specify level |
| 72040 | Radiologic examination, spine, cervical; 2 or 3 views |
| 72050 | Radiologic examination, spine, cervical; 4 or 5 views |
| 72052 | Radiologic examination, spine, cervical; 6 or more views |
| 72069 | Radiologic examination, spine, thoracolumbar, standing |
| 72070 | Radiologic examination, spine; thoracic, 2 views |
| 72072 | Radiologic examination, spine; thoracic, 3 views |
| 72074 | Radiologic examination, spine; thoracic, minimum of 4 views |
| 72080 | Radiologic examination, spine; thoracolumbar junction, minimum of 2 views |
| 72081 | Radiologic examination, spine, entire thoracic and lumbar, including skull, cervical and sacral spine if performed (e.g., scoliosis evaluation); one view |
| 72082 | Radiologic examination, spine, entire thoracic and lumbar, including skull, cervical and sacral spine if performed (e.g., scoliosis evaluation); 2 or 3 views |
| 72083 | Radiologic examination, spine, entire thoracic and lumbar, including skull, cervical and sacral spine if performed (e.g., scoliosis evaluation); 4 or 5 views |
| 72084 | Radiologic examination, spine, entire thoracic and lumbar, including skull, cervical and sacral spine if performed (e.g., scoliosis evaluation); minimum of 6 views |
| 72090 | Radiologic examination, spine; scoliosis study, including supine and erect studies |
| 72100 | Radiologic examination, spine, lumbosacral; 2 or 3 views |
| 72110 | Radiologic examination, spine, lumbosacral; minimum of 4 views |
| 72114 | Radiologic examination, spine, lumbosacral; complete, including bending views, minimum of 6 views |
| 72120 | Radiologic examination, spine, lumbosacral; bending views only, 2 or 3 views |
| 72125 | Computed tomography, cervical spine; without contrast material |
| 72126 | Computed tomography, cervical spine; with contrast material |
| 72127 | Computed tomography, cervical spine; without contrast material, followed by contrast material(s) and further sections |
| 72128 | Computed tomography, thoracic spine; without contrast material |
| 72129 | Computed tomography, thoracic spine; with contrast material |
| 72130 | Computed tomography, thoracic spine; without contrast material, followed by contrast material(s) and further sections |
| 72131 | Computed tomography, lumbar spine; without contrast material |
| 72132 | Computed tomography, lumbar spine; with contrast material |
| 72133 | Computed tomography, lumbar spine; without contrast material, followed by contrast material(s) and further sections |
| 72141 | Magnetic resonance (e.g., proton) imaging, spinal canal and contents, cervical; without contrast material |
| 72142 | Magnetic resonance (e.g., proton) imaging, spinal canal and contents, cervical; with contrast material(s) |
| 72146 | Magnetic resonance (e.g., proton) imaging, spinal canal and contents, thoracic; without contrast material |
| 72147 | Magnetic resonance (e.g., proton) imaging, spinal canal and contents, thoracic; with contrast material(s) |
| 72148 | Magnetic resonance (e.g., proton) imaging, spinal canal and contents, lumbar; without contrast material |
| 72149 | Magnetic resonance (e.g., proton) imaging, spinal canal and contents, lumbar; with contrast material(s) |
| 72156 | Magnetic resonance (e.g., proton) imaging, spinal canal and contents, without contrast material, followed by contrast material(s) and further sequences; cervical |
| 72157 | Magnetic resonance (e.g., proton) imaging, spinal canal and contents, without contrast material, followed by contrast material(s) and further sequences; thoracic |
| 72158 | Magnetic resonance (e.g., proton) imaging, spinal canal and contents, without contrast material, followed by contrast material(s) and further sequences; lumbar |
| 72159 | Magnetic resonance angiography, spinal canal and contents, with or without contrast material(s) |
| 72191 | Computed tomographic angiography, pelvis, with contrast material(s), including noncontrast images, if performed, and image postprocessing |
| 72200 | Radiologic examination, sacroiliac joints; less than 3 views |
| 72202 | Radiologic examination, sacroiliac joints; 3 or more views |
| 72220 | Radiologic examination, sacrum and coccyx, minimum of 2 views |
| 72240 | Myelography, cervical, radiological supervision and interpretation |
| 72255 | Myelography, thoracic, radiological supervision and interpretation |
| 72265 | Myelography, lumbosacral, radiological supervision and interpretation |
| 72270 | Myelography, 2 or more regions (e.g., lumbar/thoracic, cervical/thoracic, lumbar/cervical, lumbar/thoracic/cervical), radiological supervision and interpretation |
| 72275 | Epidurography, radiological supervision and interpretation |
| 72285 | Discography, cervical or thoracic, radiological supervision and interpretation |
| 72295 | Discography, lumbar, radiological supervision and interpretation |
| 77072 | Bone age studies |
| 77075 | Radiologic examination, osseous survey; complete (axial and appendicular skeleton) |
| 77078 | Computed tomography, bone mineral density study, 1 or more sites, axial skeleton (e.g., hips, pelvis, spine) |
| 77080 | Dual-energy X-ray absorptiometry (DXA), bone density study, 1 or more sites; axial skeleton (e.g., hips, pelvis, spine) |
| 77084 | Magnetic resonance (e.g., proton) imaging, bone marrow blood supply |
| 77085 | Dual-energy X-ray absorptiometry (DXA), bone density study, 1 or more sites; axial skeleton (e.g., hips, pelvis, spine), including vertebral fracture assessment |
| 77086 | Vertebral fracture assessment via dual-energy X-ray absorptiometry (DXA) |

**Appendix 2**: Number and prevalence of enrollees with spine degeneration by age group and sex

**Appendix 3**: Number and prevalence of enrollees with stenosis by age group and sex.

**Appendix 4**: Number and prevalence of enrollees with spine curvature by age group and sex.

**Appendix 5**: Number and prevalence of enrollees with DISH by age group and sex.

**Appendix 6**: Number and prevalence of enrollees with spondylitis by age group and sex.

**Appendix 7**: Number and prevalence of enrollees with disc disease by age group and sex.

**Appendix 8**: Number and prevalence of enrollees with osteoporosis by age group and sex.

**Appendix 9**: Number and prevalence of enrollees with other types of spine degeneration by age group and sex.

**Appendix 10**: Number and frequency of enrollees by spine degeneration type, age group, obesity coding, and gender.

**Appendix 11**: Number and frequency of enrollees with radiology by spine degeneration type, age group, and gender.

**Appendix 12:** Additional discussion of spine degeneration prevalence and comorbidities reported in the literature.

The prevalence of degenerative spine pathologies in older individuals is important to understanding their overall health and injury risk, as well as evaluating whether identified pathologies are acute or the result of degenerative processes. Cheng et al. (2014) reported that individuals 65 and older comprise about 39% of the total US population. Table A12-1 shows a summary of prevalence of specific spine degenerative pathology reported in the literature.

Table A12-1 is followed by additional discussion of degenerative spinal pathologies considered in this analysis, including relevant comorbidities and sequelae. References are cited in context; additional references, including those from Table A12-1, are provided at the end of this Appendix 12.

Table A12-1: Selected prevalence of specific spine degenerative pathology.

Table A12-1: Selected prevalence of specific spine degenerative pathology (cont.).

**Stenosis**

Stenosis refers to narrowing of the spinal canal or neural foramina. Stenosis can be congenital but is commonly a degenerative finding. Stenosis is often assessed using the Torg or the Torg-Pavlov ratio, the diameter of the spinal canal divided by the diameter of the vertebral body.^35, 36, 37^

The increase in stenosis prevalence with age is well documented in the literature.^15, 16, 17^ Vogt et al. (2006) analyzed U.S. census data and estimated that 2.45 million older men and 3.54 million older women had symptoms suggestive of either cervical or lumbar stenosis.^54^ They estimated that these numbers would more than double from 6 million in 2000 to 14.9 million in 2050.

Shim et al. (2009) reported a prevalence of 4% to 9% in the cervical spine based on cadaveric data.^29^ Wang et al. (2019) reported a prevalence in individuals age 74 and older of 38.6% for Grade 1 stenosis, defined as ≥50% narrowing of the anterior subarachnoid space without spinal cord compression.^30^

Yabuki et al. (2013) estimated a prevalence of 5.7% for stenosis in the lumbar spine.^55^ The prevalence of acquired (degenerative) stenosis in the lumbar spine has been reported to increase with age and body mass index (BMI).^32, 33^ Kalichman et al. (2009) reported that the prevalence of lumbar spine stenosis was similar for males and females, and for asymptomatic and symptomatic individuals.^32^

Stenosis can increase the risk of neurological injuries in traumatic events. Takao et al. (2013) reported that the risk of traumatic cervical spine injury was 124.5-times greater in individuals with cervical spinal canal stenosis than without. The association between cervical stenosis and disabling spinal cord injury is well documented in the literature.^46, 47, 48^

**Spondylitis**

Spondylitis is a general term referring to inflammation of the joints of the spine. Inflammation may result when ligaments, tendons, or joints of the spine are acutely injured. Inflammation of the spine may be associated with systemic inflammatory diseases, such as rheumatoid arthritis (RA) or lupus erythematosus. Inflammation may be a chronic response to repetitive loading (wear and tear), especially if the mechanical environment is altered due to other degenerative processes, such as decreased disc height or spine curvature. Fredrickson et al. (1984) conducted of roentgenographic study and reported that the prevalence of spondylolysis was 4.4% in children and 6% of the adult population.^56^ Inflammation has been implicated as a cause of lumbar pain in obese individuals in addition to the increased mechanical loading associated with the excess weight.^20^

One type of spondylitis with notable biomechanical significance is Ankylosing Spondylitis (AS). AS is a chronic rheumatic disease that can cause inflammation in the sacroiliac joints and in vertebrae and spinal ligaments. Also known as Bechterew’s disease, AS initially manifests in the lower back but can spread into the cervical spine. AS results in increased spine stiffness.

AS is associated with an increased risk of vertebral fracture.^57-60^ While the prevalence of AS overall is low, the prevalence of osteoporosis and vertebral fractures within this group is high at a relatively young age.^61^ The prevalence of osteoporosis and osteopenia in the lumbar spine of AS patients has been reported to be 16% and 39%, respectively, within the first decade of diagnosis. Klingberg et al. (2012) summarized that AS involves distinct and opposite bone remodelling processes: new bone formation in the cortical zone of the vertebrae and ligaments, and loss of trabecular bone in the vertebral body.^62^ The increased prevalence of osteoporosis in AS patients can be obscured by the characteristic formation of syndesmophytes that may artificially raise BMD quantification in certain assessment protocols.^62^ Montala et al. (2011) reported a prevalence of vertebral fractures of 32.4% in AS patients, most were mild and localized in the thoracic spine. ^63^

**Spine Curvatures (kyphosis, lordosis, and scoliosis)**

Age-related kyphosis is colloquially referred to as Dowager’s hump or gibbous deformity.^64^ Kyphosis is often associated with wedge-shaped compression of the thoracic vertebrae secondary to osteoporosis. Kyphosis can also be a sequela of ankylosing spondylitis (AS). Kyphosis may also develop secondary to poor posture and muscle weakness. Kyphosis is quantified using the Cobb angle or Kyphosis Index (KI). Scoliosis is typically quantified using the Cobb angle and may be additionally quantified by the angle of pelvic tilt as well as the sagittal and coronal balances.

Kyphosis has been specifically associated with increased spine curvature with age.^13, 14, 15^ Parenteau et al. (2020) reported a prevalence of 0.6% in trauma registry individuals.^15^ Katzman et al. (2010) showed a prevalence of approximately 20% to 40% in older adults based on a literature review.^64^

Holcombe et al. (2017) showed how the spine kyphotic curvature increased in older females.^14^ Excessive kyphosis can also occur in infants or teens due to malformation of the spine or wedging of the spinal bones. Kyphosis can alter the loading pattern and affect the injury outcome. Kyphosis can result in degenerative changes, vertebral compression fractures, muscular weakness, and altered biomechanics. Injury can occur during thoracic spine straightening in extension, even with minor trauma.^42, 43^

**Diffuse Idiopathic Skeletal Hyperostosis (DISH)**

DISH is generally identified using Resnick’s diagnosis criteria,^38^ which consist of a) the presence of right-sided, flowing, coarse osteophytes in the thoracic spine connecting at least four contiguous vertebrae or ossification of the anterior longitudinal ligament, b) preserved intervertebral disc height in the involved segment and c) the absence of apophyseal joint ankylosis and sacroiliac joint involvement.

The prevalence of DISH ranges from 4% to 35% across different study populations and diagnostic criteria used. DISH is observed mostly in elderly with a male preponderance. Kiss et al. (2002) found 27.3% of men and 12.8% of women older than 50 years old had evidence of DISH.^25^ Uehara et al (2020) reported a prevalence of 17.5% in a population based study of Japanese men and women age 50 and older.^27^ It is notable that the prevalence increased with age from 3.1% for those in their 50s to 14.0% in their 60s, 24.3% in their 70s and 29.0% in their 80s.

The prevalence of DISH was up to 25% to 35% in individuals older than 70.^24, 27^ Boachie-Adjei et al. (1987) investigated 75 autopsy reports and reported a prevalence up to 28%.^4^ Weinfield et al. (1997) reviewed more than 2,300 chest radiographs and reported a prevalence of 35% in males 71 and older.^24^

The spinal involvement of DISH has long been considered a radiographic entity with minor and nonsignificant clinical manifestations. Patients with DISH can have marked limitations of spinal mobility and occasionally may have postural abnormalities and spinal pain.^65^ Peripheral enthesopathy of DISH is usually not painful. Forestier and Rotes-Querol (1950) noted that severe trauma occurred in a third of their cases and may have been the precipitating cause leading to the diagnosis of DISH.^66^

Diederichs et al. (2010) found that DISH was associated with a higher prevalence of vertebral fractures in elderly men.^67^ Fused segments resulting from DISH or other ossification can become brittle and more prone to fracture in relatively minor trauma.^53^ Individuals with DISH have been reported to have higher incidence of traumatic vertebral fracture and are more likely to have multiple fractures.^52^ However, Paley et al. (1991) noted that spine fractures involving DISH are uncommonly reported, and a diagnosis of DISH may be delayed.^68^

The pathogenesis of DISH involves several risk factors based on association with various metabolic conditions. Some of these factors include insulin-dependent diabetes mellitus, obesity, gout, high uric acid and dyslipidemia.^25, 69-71^

**Disc degeneration**

Boden et al. (1990) analyzed MRI data and indicated that 80% of asymptomatic patients 60 years and older had cervical disc degeneration.^28^ Since then, numerous studies have been performed to assess prevalence of spinal degeneration. Brinjikji et al. (2015) published results of a systematic literature review that included 33 articles reporting imaging findings in asymptomatic individuals.^7^ The prevalence of disc degeneration was reported to increase from 37% in 20-29 year-olds to 96% of individuals 80 and older; prevalence was further reported for specific types of degeneration including disc bulge, disc protrusion, and annular fissures. Table 1 summarizes some of the findings.

Samartzis et al. (2012) reported that disc degeneration was 30% more likely in overweight individuals and 79% more likely in obese individuals than healthy-weight individuals.^21^ The authors also reported that end stage disc degeneration with disc space narrowing was 72% more likely in obese subjects. The effects of increased weight on spine degeneration appear to be time-dependent, as might be expected.

The association between age and disc degeneration is well documented in the literature.^7, 8^ Liuke et al. (2005) reported that middle-aged overweight men were more likely to have a greater number of degenerated discs in the lumbar spine if they had also been overweight as younger adults than if their weight gain occurred later in life.^72^ The distribution of extra weight also affects the load on the spine and resulting risk of degeneration.

**Osteoporosis**

Based on 2013-2014 U.S. National Health and Nutrition Examination Survey (NHANES) data, Looker et al. (2017) reported that 8% of adults older than 50 years had osteoporosis.^19^ Looker et al. also reported a much higher prevalence in females (11.6%) compared to males (3.6%). Some studies have reported much higher prevalences for certain demographic groups. Looker et al. further examined the 2013-2014 NHANES data for differences in prevalence of osteoporosis by race and reported that non-Hispanic Asian women had the highest prevalence at 38.8%. Shetty et al. (2020) reported that about 30% of 400 post-menopausal Indian women who were referred for assessment of bone mineral density had osteoporosis.^73^ Based on earlier NHANES data from 2005-2008, Wright et al. reported a prevalence of osteoporosis of 30% in women age 50 or more, and a much higher prevalence of 77% in women age 80 or more.^74^

The risk of vertebral fractures is associated with lower bone density.^75-81^ Legrand et al. (1999) found a one standard deviation drop in spinal bone mineral density (BMD) was associated with a 2.7-fold increase in the risk of vertebral fracture.^81^ The study confirmed the association of age and BMD with vertebral fractures in middle-aged men.

Rostom et al. (2012) reported a much higher prevalence of thoracic and lumbar vertebral fractures in postmenopausal women and reported an association with osteoporosis.^82^ Shetty et al. (2020) reported that nearly 2 out of 3 post-menopausal Indian women who were referred for assessment of bone mineral density had existing vertebral fractures.^73^ They reported that about half of those with vertebral fractures had osteoporosis (about 1 in 3 overall). Li et al. (2018) evaluated the radiology of 3216 Chinese female patients age 50 and older and reported the prevalence of thoracic vertebral fracture was 2.4% in those age 50-59, 8.9% in those age 60-69 years, and 21.9% in those 70 or older.^53^ The authors noted that two-thirds of the patients had been undiagnosed in the original radiology reports.

Other disorders including Rheumatoid Arthritis (RA) and systemic lupus erythematosus (SLE) are associated with increased risk of osteoporosis.^83^ Ghazi et al. (2011) reported that women with RA had an increased risk of vertebral fractures (odds ratio 6.5).^84^ The risk of vertebral fractures associated with RA has been reported to increase with the duration and severity of the RA.^85, 86^ In a case-control study, prevalence of osteoporosis in women with RA was 55.4% vs. 10.5% in controls.^84^ AS and RA have been reported to be associated with spine degeneration at a younger age,^61^ which cannot be identified with the Medicare data due to the minimum age of 65. Inactivity due to pain associated with these diseases further contributes to bone loss. A recent case-control study of patients with chronic obstructive pulmonary disease (COPD) found that the COPD group had lower BMD and 2.6 times greater odds of osteoporosis.^87^ Silva et al. (2011) previously reported a prevalence of osteoporosis or osteopenia of 84% (42% each) in a cross-sectional study of 95 men and women (mean age 67) with COPD.^88^ Glucocorticoid use to treat systemic inflammatory disorders can further exacerbate bone loss if not mitigated.^89^

**Spine Degeneration and Vertebral Fractures**

As discussed above, specific degenerative spine pathologies are associated with increased risk of vertebral fractures. There are differences in reported fracture patterns between the types of spine degeneration. These differences may inform whether a specific degenerative spinal pathology may have contributed to an injury mechanism. Osteoporosis is more often associated with vertebral compression fractures. Shetty et al. (2020) reported that osteoporosis-related fractures were predominately located in the lower thoracic spine and showed crush, wedge, and/or biconcave characteristics.^73^ Paley et al. (1991) reported that two fracture patterns associated with DISH were observed.^68^ One type was found through the midportion of an ankylosed segment of the spine and involved the vertebral body. The second type occurred at the top or bottom of a fused segment and involved disc disruptions or odontoid fractures. Fractures associated with AS more commonly either extend through the disc (intradiscal) or are located at or near the vertebral endplate and include the posterior elements.^57, 58, 60^

**Additional References**

1. Vogt MT, Cawthon PM, Kang JD, Donaldson WF, Cauley JA, Nevitt MC. Prevalence of symptoms of cervical and lumbar stenosis among participants in the Osteoporotic Fractures in Men Study. *Spine*. 2006;31(13):1445-1451.
2. Yabuki S, Fukumori N, Takegami M, et al. Prevalence of lumbar spinal stenosis, using the diagnostic support tool, and correlated factors in Japan: a population-based study. *J Orthop Sci*. 2013;18(6):893-900.
3. Fredrickson BE, Baker D, McHolick WJ, et al. The natural history of spondylolysis and spondylolisthesis. *J Bone Joint Surg Am*. 1984;66:699–707.
4. Gelman MI, Umber JS. Fractures of the thoracolumbar spine in ankylosing spondylitis. *Am J Roentgen*. 1978;130(3):485-491.
5. Murray GC, Persellin RH. Cervical fracture complicating ankylosing spondylitis: a report of eight cases and review of the literature. *Am J Med*. 1981;70(5):1033-1041.
6. Werner BC, Samartzis D, Shen FH. Spinal fractures in patients with ankylosing spondylitis: etiology, diagnosis, and management. *JAAOS*. 2016;24(4):241-249.
7. Weinstein PR, Karpman RR, Gall EP, Pitt M. Spinal cord injury, spinal fracture, and spinal stenosis in ankylosing spondylitis. *J Neurosurg*. 1982;57(5):609-616.
8. van der Weijden M, Claushuis TA, Nazari T, Lems WF, Dijkmans BA, Van Der Horst-Bruinsma IE. High prevalence of low bone mineral density in patients within 10 years of onset of ankylosing spondylitis: a systematic review. *Clin Rheumatol*. 2012;31(11):1529-35.
9. Klingberg E, Lorentzon M, Mellström D, et al. Osteoporosis in ankylosing spondylitis-prevalence, risk factors and methods of assessment. *Arthritis Res Ther*. 2012;14(3):R108.
10. Montala N, Juanola X, Collantes E, et al. Prevalence of vertebral fractures by semiautomated morphometry in patients with ankylosing spondylitis. *J Rheum*. 2011;38(5):893-897.
11. Katzman WB, Wanek IL, Shepherd JA, Sellmeyer DE. Age-related hyperkyphosis: its causes, consequences, and management. *J Orthop Sports Phys Ther*. 2010;40(6):352–360. doi: 0.2519/jospt.2010.3099
12. Mata S, Fortin PR, Fitzcharles MA, et al. A controlled study of diffuse idiopathic skeletal hyperostosis. Clinical features and functional status. *Medicine*. 1997;76(2):104-117.
13. Forestier J, Rotés-Querol J. Senile ankylosing hyperostosis of the spine. *Ann Rheum Dis*. 1950;9(4):321.
14. Diederichs G, Engelken F, Marshall LM, et al. Osteoporotic fractures in men (MrOS) research group. Diffuse idiopathic skeletal hyperostosis (DISH): relation to vertebral fractures and bone density. *Osteoporos Int*. 2011;22(6):1789-1797.
15. Paley D, Schwartz M, Cooper P, Harris WR, Levine AM. Fractures of the spine in diffuse idiopathic skeletal hyperostosis. *Clin Orthop Rel Res*. 1991;267:22-32.
16. Littlejohn GO, Smythe HA. Marked hyperinsulinemia after glucose challenge in patients with diffuse idiopathic skeletal hyperostosis. *Journal Rheum*. 1981;8(6):965-8.
17. Littlejohn GO, Urowitz MB. Peripheral enthesopathy in diffuse idiopathic skeletal hyperostosis (DISH): a radiologic study. *Journal Rheum*. 1982;9(4):568-72.
18. Vezyroglu G, Mitropoulos A, Kyriazis N, Antoniadis C. A metabolic syndrome in diffuse idiopathic skeletal hyperostosis: a controlled study. *J Rheumatol*. 1996;23:672-676.
19. Liuke M, Solovieva S, Lamminen A. Disc degeneration of the lumbar spine in relation to overweight. *Int J Obes*. 2005;29(8):903-8.
20. Shetty S, John B, Mohan S, Paul TV. Vertebral fracture assessment by dual-energy X-ray absorptiometry along with bone mineral density in the evaluation of postmenopausal osteoporosis. *Arch Osteoporos*. 2020;15(1):1-6.
21. Wright NC, Saag KG, Dawson-Hughes B, Khosla S, Siris ES. The impact of the new National Bone Health Alliance (NBHA) diagnostic criteria on the prevalence of osteoporosis in the USA. *Osteoporos Int*. 2017;28(4):1225-1232.
22. Melton III LJ, Kan SH, Frye MA, Wahner HW, O'fallon WM, Riggs BL. Epidemiology of vertebral fractures in women. *Am J Epid*. 1989;129(5):1000-1011.
23. Mosekilde L. Sex differences in age-related loss of vertebral trabecular bone mass and structure—biomechanical consequences. *Bone*. 1989;10(6):425-32.
24. Stone KL, Seeley DG, Lui LY, et al. BMD at multiple sites and risk of fracture of multiple types: long‐term results from the Study of Osteoporotic Fractures. *J Bone Miner Res*. 2003;18(11):1947-1954.
25. Ruyssen-Witrand A, Gossec L, Kolta S, Dougados M, Roux C. Vertebral dimensions as risk factor of vertebral fracture in osteoporotic patients: a systematic literature review. *Osteoporos Int*. 2007;18(9):1271-8.
26. Arboleya L, Díaz-Curiel M, Del Río L, et al. Prevalence of vertebral fracture in postmenopausal women with lumbar osteopenia using MorphoXpressSR (OSTEOXPRESS Study). *Aging Clin Exper Res*. 2010;22(5-6):419-426.
27. Jacques RM, Boonen S, Cosman F, et al. Relationship of changes in total hip bone mineral density to vertebral and nonvertebral fracture risk in women with postmenopausal osteoporosis treated with once‐yearly zoledronic acid 5 mg: the HORIZON‐Pivotal Fracture Trial (PFT). *J Bone Miner Res.* 2012;27(8):1627-1634.
28. Legrand E, Chappard D, Pascaretti C, et al. Bone mineral density and vertebral fractures in men. *Osteoporosis Int.* 1999;10(4):265-270.
29. Rostom S, Allali F, Bennani L, Abouqal R, Hajjaj-Hassouni N. The prevalence of vertebral fractures and health-related quality of life in postmenopausal women. *Rheum Int.* 2012;32(4):971-980.
30. Bultink IE, Vis M, van der Horst-Bruinsma IE, Lems WF. Inflammatory rheumatic disorders and bone. *Curr Rheum Rep*. 2012;14(3):224-230.
31. Ghazi M, Kolta S, Briot K, Fechtenbaum J, Paternotte S, Roux C. Prevalence of vertebral fractures in patients with rheumatoid arthritis: revisiting the role of glucocorticoids. *Osteoporosis Int.* 2012;23(2):581-587.
32. El Maghraoui A, Rezqi A, Mounach A, Achemlal L, Bezza A, Ghozlani I. Prevalence and risk factors of vertebral fractures in women with rheumatoid arthritis using vertebral fracture assessment. *Rheumatology*. 2010;49(7):1303-1310.
33. Mohammad A, Lohan D, Bergin D, et al. The prevalence of vertebral fracture on vertebral fracture assessment imaging in a large cohort of patients with rheumatoid arthritis. *Rheumatology*. 2014;53(5):821-827.
34. Adas-Okuma MG, Maeda SS, Gazzotti MR, et al. COPD as an independent risk factor for osteoporosis and fractures. *Osteoporos Int*. 2019:1-1.
35. Silva DR, Coelho AC, Dumke A, et al. Osteoporosis prevalence and associated factors in patients with COPD: a cross-sectional study. *Resp Care*. 2011;56(7):961-968
36. Weinstein RS. Glucocorticoid-induced bone disease. *NEJM*. 2011;365(1):62-70.
37. Anagnostopoulos I, Zinzaras E, Alexiou I et al. The prevalence of rheumatic diseases in central Greece: a population survey. *BMC Musculoskelet Disord*. 2010;11:98105.
38. Ballane G, Cauley JA, Luckey MM, Fuleihan GE. Worldwide prevalence and incidence of osteoporotic vertebral fractures. *Osteoporos Int*. 2017;28(5):1531-42.
39. Bilston LE, Clarke EC, Brown J. Spinal injury in car crashes: crash factors and the effects of occupant age. *Inj Prev*. 2011;17(4): 228-32.
40. Blackmore CC, Ramsey SD, Mann FA, Deyo RA. Cervical spine screening with CT in trauma patients: a cost-effectiveness analysis. *Emerg Radiol*. 1999;212(1) Published online: Jul 1 1999 doi:10.1148/radiology.212.1.r99jl08117
41. Bloom RA. The prevalence of ankylosing hyperostosis in a Jerusalem population: with description of a method of grading the extent of the disease. *Scand J Rheumatol*. 1984;13:181–189.
42. Carter OD, Haynes S. Prevalence rates for scoliosis in US adults: results from the first national health and nutrition examination survey. *Int J Epidemiol*. 1987;16:537–544. doi: 10.1093/ije/16.4.537
43. Cutler WB, Friedmann E, Genovese-Stone E. Prevalence of kyphosis in a healthy sample of pre- and postmenopausal women. *Am J Phys Med Rehabil*. 1993;72:219-225.
44. Damborg F, Engell V, Andersen M, Kyvik KO, Thomsen K. Prevalence, concordance, and heritability of Scheuermann kyphosis based on a study of twins. *J Bone Joint Surg Am*. 2006;88(10):2133-2136.
45. Dean LE , Jones GT , MacDonald AG, Downham C, Sturrock RD, Macfarlane GJ. Global prevalence of ankylosing spondylitis. *Rheumatol*. 2014;53:650-657.
46. Epstein N. Ossification of the cervical posterior longitudinal ligament: a review. *Neurosurg Focus*. 2002;13(2):1-10.
47. Epstein NE. The surgical management of ossification of the posterior longitudinal ligament in 43 North Americans. *Spine*. 1994;19:664-672.
48. Epstein NE. What you need to know about ossification of the posterior longitudinal ligament to optimize cervical spine surgery: A review. *Surg Neurol Int*. 2014;5(Suppl 3):S93-S118.
49. Ernst CW, Stadnik TW, Peeters E, Breucq C, Osteaux MJ. Prevalence of annular tears and disc herniations on MR images of the cervical spine in symptom free volunteers. *Eur J Radiol*. 2005;55(3):409-14.
50. Fabreguet I, Fechtenbaum J, Briot K, Paternotte S, Roux C. Lumbar disc degeneration in osteoporotic men: prevalence and assessment of the relation with presence of vertebral fracture. *J Rheumatol*. 2013;40(7):1183-90.
51. Friedenberg ZB, Miller WT. Degenerative disc disease of the cervical spine: a comparative study of asymptomatic and symptomatic patients. *J Bone Joint Surg Am*. 1963;45(6):1171-1178.
52. Fujimori T, Watabe T, Iwamoto Y, et al. Prevalence, concomitance, and distribution of ossification of the spinal ligaments: results of whole spine CT scans in 1500 Japanese patients. *Spine*. 2016;41:1668-1676.
53. Geusens P, De Winter L, Quaden D, et al. The prevalence of vertebral fractures in spondyloarthritis: relation to disease characteristics, bone mineral density, syndesmophytes and history of back pain and trauma. *Arthritis Res Ther*. 2015;17(1):294.
54. Gore DR, Sepic SB, Gardner GM. Roentgenographic findings of the cervical spine in asymptomatic people. *Spine*. 1986;11(6):521-524.
55. Gore DR. Roentgenographic findings in the cervical spine in asymptomatic persons: a ten-year follow-up. *Spine*. 2001;26(22):2463-2466.
56. Graffy PM, Lee SJ, Ziemlewicz TJ, Pickhardt PJ. Prevalence of vertebral compression fractures on routine CT scans according to L1 trabecular attenuation: determining relevant thresholds for opportunistic osteoporosis screening. *AJR Am J Roentgenol*. 2017;209(3):491-496.
57. Greaves LL; Van Toen C, Melnyk A, Koenig L, Zhu Q, Tredwell S ; Mulpuri K, Cripton, PA. Pediatric and adult three-dimensional cervical spine kinematics: effect of age and sex through overall motion. *Spine*. 2009;34(16):1650-1657. doi: 10.1097/BRS.0b013e3181ab65c6
58. Hales CM, Carroll MD, Fryar CD, Ogden CL. Prevalence of obesity among adults and youth: United States, 2015–2016. U.S. Department of Health and Human Services, NCHS Data Brief No. 288, October, 2017.
59. Hasserius R, Karlsson MK, Nilsson BE et al. Prevalent vertebral deformities predict increased mortality and increased fracture rate in both men and women: a 10-year population-based study of 598 individuals from the Swedish cohort in the European Vertebral Osteoporosis Study. *Osteoporos Int*. 2003;14:61–68.
60. Hong JY, S W Suh, H N Modi, C Y Hur, H R Song, J H Park. The prevalence and radiological findings in 1347 elderly patients with scoliosis. *J Bone Joint Surg Br*. 2010;92(7):980-983. doi: 10.1302/0301-620X.92B7.23331
61. Ishimoto Y, Yoshimura N, Muraki S, et al. Prevalence of symptomatic lumbar spinal stenosis and its association with physical performance in a population-based cohort in Japan: the Wakayama Spine Study. *Osteoarthr Cartil*. 2012;20(10):1103-1108.
62. Jansz TT, Goto NA, van Ballegooijen AJ, Willems HC, Verhaar MC, van Jaarsveld BC. The prevalence and incidence of vertebral fractures in end-stage renal disease and the role of parathyroid hormone. *Osteoporos Int*. 2020;31(3):515-524.
63. Jensen MC, Brant-Zawadzki MN, Obuchowski N, Modic MT, Malkasian D, Ross JS. Magnetic resonance imaging of the lumbar spine in people without back pain. *N Engl J Med*. 1994;331(2):69-73.
64. Julkunen H, Heinonen OP, Knekt P *et al.* The epidemiology of hyperostosis of the spine together with its symptoms and related mortality in a general population. *Scand J Rheumatol*. 1975;4:23-27.
65. Julkunen H, Heinonen OP, Pyorala K. Hyperostosis of the spine in an adult population. Its relation to hyperglycaemia and obesity. *Ann Rheum Dis*. 1971;30:605-612.
66. Julkunen H, Knekt P, Aromaa A. Spondylosis deformans and diffuse idiopathic skeletal hyperostosis (DISH) in Finland. *Scand J Rheumatol*. 1981;10:193-203.
67. Kado DM, Huang MH, Karlamangla AS, Barrett‐Connor E, Greendale GA. Hyperkyphotic posture predicts mortality in older community‐dwelling men and women: a prospective study. *J Am Geriatr Soc*. 2004;52(10):1662-1667.
68. Kaipiainen-Seppanen O, Aho K, Heliovaara M. Incidence and prevalence of ankylosing spondylitis in Finland. *J Rheumatol*. 1997;24:4969.
69. Kalanithi PA, Arrigo R, Boakye M. Morbid obesity increases cost and complication rates in spinal arthrodesis. *Spine*. 2012;37(11):982-988. doi: 10.1097/BRS.0b013e31823
70. Katogoni R, Yoshida M, Muraki S, Oka H, Hashizume H, et al. Prevalence of diffuse idiopathic skeletal hyperostosis (DISH) of the whole spine and its association with lumbar spondylosis and knee osteoarthritis: the ROAD study. *J Bone Miner Metab*. 2015;33: 221–229.
71. Key CA. Paraplegia depending on disease of the ligaments of the spine. *Guys Hosp Rep*. 1938;3:17–34.
72. Koyanagi HI, Fujimoto S, Hida K, Iwasaki Y, Houkin K. Spinal canal size in ossification of the posterior longitudinal ligament of the cervical spine. *Surg Neurol*. 2004;62(4):286-291.
73. Koyanagi I, Iwasaki Y, Hida K, Akino M, Imamura H, Abe H. Acute cervical cord injury without fracture or dislocation of the spinal column. *J Neurosurg*. 2000;93(1 Suppl):15-20.
74. Majumdar SR, Villa-Roel C, Lyons KJ, Rowe BH. Prevalence and predictors of vertebral fracture in patients with chronic obstructive pulmonary disease. *Respir Med*. 2010;104(2):260-266.
75. Marquez-Lara A, Nandyala SV, Fineberg, SJ. Singh K. Current trends in demographics, practice, and in-hospital outcomes in cervical spine surgery: a national database analysis between 2002 and 2011. *Spine*. 2014;39(6):476-481. doi: 10.1097/BRS.0000000000000165
76. Masashi N, Takeshi S, Shunsuke F, Takashi N. The clinical risk of vertebral artery injury from cervical pedicle screws inserted in degenerative vertebrae. *Spine*. 2005;30(24):2800-2805. doi: 10.1097/01.brs.0000192297.07709.5d
77. Matsumoto M, Fujimura Y, Suzuki N, et al. MRI of cervical intervertebral discs in asymptomatic subjects. *J Bone Joint Surg Br*. 1998;80(1):19-24.
78. Mikkelsen WM. Estimates of the prevalence of rheumatic diseases in the population of Tecumseh, Michigan, 1959‑60. *J Chronic Dis*. 1967;20:351-369.
79. Miller JA, Schmatz C, Schultz AB. Lumbar disc degeneration: correlation with age, sex, and spine level in 600 autopsy specimens. *Spine*. 1988;13(2):173-178.
80. Mori K, Kasahara T, Mimura T, Nishizawa K, Nakamura A, Imai S. Prevalence of thoracic diffuse idiopathic skeletal hyperostosis (DISH) in Japanese: results of chest CT-based cross-sectional study. *J Orthop Sci*. 2017;22:38–42.
81. Morishita Y, Naito M, Hymanson H, Miyazaki M, Wu G, Wang JC. The relationship between the cervical spinal canal diameter and the pathological changes in the cervical spine. *Eur Spine J*. 2009;18:877–883.
82. Morishita Y, Naito M, Wang JC. Cervical spinal canal stenosis: the differences between stenosis at the lower cervical and multiple segment levels. *Int Orthop*. 2011;35:1517–1522.
83. Nagata K, Yoshimura N, Hashizume H, et al. The prevalence of tandem spinal stenosis and its characteristics in a population-based MRI study: The Wakayama Spine Study. *Eur Spine J*. 2017;26(10):2529-2535.
84. Nascimento FA, Gatto LA, Lages RO, Neto HM, Demartini Z, Koppe GL. Diffuse idiopathic skeletal hyperostosis: a review. *Surg Neurol Int*. 2014;5(Suppl 3):S122–S125.
85. Ognjenovic M, Raymond W, Inderjeeth C, Keen H, Preen D, Nossent J. The risk and consequences of vertebral fracture in patients with Ankylosing Spondylitis: a population-based data linkage study. *J* *Rheumatol*. 2020; doi: 10.3899/jrheum.190675
86. Ohtsuka K, Terayama K, Yanagihara M, et al. An epidemiological survey on ossification of ligaments on the cervical and thoracic spine on individuals over 50 years of age. *J Jpn Orthop Assoc*. 1986;60:1087–1098.
87. Pappone N, Lubrano E, Esposito-del PA, et al. Prevalence of diffuse idiopathic skeletal hyperostosis in a female Italian population. *Clin Exp Rheumatol*. 2005;23:123-124.
88. Parenteau CS, Viano DC. Spinal fracture-dislocations and spinal cord injuries in motor vehicle crashes. *Traffic Inj Prev*. 2014;15(7):694-700. doi: 10.1080/15389588.2013.867434.
89. Parenteau CS, Wang N, Wang S. Quantification of pediatric and adult cervical vertebra: anatomical characteristics by age and gender for automotive application. *Traffic Inj Prev*. 2014;15(6):572-582.
90. Parenteau CS, Zhang P, Holcombe S, Wang S. Characterization of vertebral angle and torso depth by gender and age groups with a focus on occupant safety. *Traffic Inj Prev*. 2014;15(1):66-72. doi: 10.1080/15389588.2013.829217.
91. Pintar FA, Yoganandan N, Maiman DJ. Thoracolumbar spine fractures in frontal impact crashes. *Ann Adv Automotive Med*. 2012;56:277–283.
92. Poncelet AN, Rose-Innes Patil PG, Turner DA, Pietrobon R. National trends in surgical procedures for degenerative cervical spine disease: 1990–2000. *Neurosurgery*. 2005;57(4):753–758. doi:10.1227/01.NEU.0000175729.79119.1d
93. Santanello SA, Falcone R, Poka A, Johnson J. Incomplete quadriplegia resulting from minor trauma: initial presentation of ossification of the posterior longitudinal ligament. *J Trauma*. 2001;50(3):578-580.
94. Saraux A, Guillemin F, Guggenbuhl P, et al. Prevalence of spondyloarthropathies in France: 2001. *Ann Rheum Dis*. 2005;64:14315.
95. Schwab F, Ashok D, Lorenzo G, et al. Adult scoliosis: prevalence, SF-36, and nutritional parameters in an elderly volunteer population. *Spine*. 2005;30:1083–1085.
96. Sekhon LHS, Fehlings MG. Epidemiology, demographics, and pathophysiology of acute spinal cord injury. *Spine*. 2001;26(245):S2–S12.
97. Sencan D, Elden H, Nacitarhan V, et al. The prevalence of diffuse idiopathic skeletal hyperostosis in patients with diabetes mellitus. *Rheumatol Int.* 2005;25:518–521. doi: 10.1007/s00296-004-0474-9
98. Shin J, Kim YW, Lee SG, Park EC, Yoon SY. Cohort study of cervical ossification of posterior longitudinal ligament in a Korean populations: demographics of prevalence, surgical treatment, and disability. *Clin Neurol Neurosurg*. 2018;166:4-9.
99. Sirasanagandla SR, Al Dhuhli H, Al Abri A, Salmi A, Jayapal SK, Sara C, Jaju S. Prevalence of diffuse idiopathic skeletal hyperostosis among elderly subjects referred for radiological investigation in tertiary hospital at Oman**.** *Anat Cell Biol*. 2018;51(3):174–179.
100. Stadnik TW, Lee RR, Coen HL, Neirynck EC, Buisseret TS, Osteaux MJ. Annular tears and disk herniation: prevalence and contrast enhancement on MR images in the absence of low back pain or sciatica. *Radiology*. 1998;206(1):49-55.
101. Takahashi T, Ishida K, Hirose D, et al. Trunk deformity is associated with a reduction in outdoor activities of daily living and life satisfaction in community-dwelling older people. *Osteoporos Int*. 2005;16(3):273-9.
102. Tang L, Zheng J, Hu J. A numerical investigation of factors affecting lumbar spine injuries in frontal crashes. *Accid Anal Prev.* 2019;136:105400. doi: 10.1016/j.aap.2019.105400. [Epub ahead of print]
103. Teraguchi M, Yoshimura N, Hashizume H, et al. Prevalence and distribution of intervertebral disc degeneration over the entire spine in a population-based cohort: the Wakayama Spine Study. *Osteoarthr Cartil*. 2014;22(1):104-110.
104. Teresi LM, Lufkin RB, Reicher MA, et al. Asymptomatic degenerative disk disease and spondylosis of the cervical spine: MR imaging. *Radiology*. 1987;164(1):83-88.
105. Tsuyama N. The ossification of the posterior longitudinal ligament (OPLL). The investigation committee on OPLL of the Japanese Ministry of Public Health and Welfare. *J Jpn Orthop Assoc*. 1981;55:425-440.
106. Tsuyama N, Teryama K, Okene K, et al. The ossification of the posterior longitudinal ligament of the spine (OPLL). *J Jpn Orthop Assoc*. 1981;55:425-440.
107. United States Bone and Joint Initiative: The Burden of Musculoskeletal Diseases in the United States (BMUS), Fourth Edition, 2018. Rosemont, IL. Available at [http://www.boneandjointburden.org](http://www.boneandjointburden.org/). Accessed on March 16, 2020.
108. Urrutia J, Zamora T, Klaber I. Thoracic scoliosis prevalence in patients 50 years or older and its relationship with age, sex, and thoracic kyphosis. *Spine*. 2014;39(2):149-152.
109. Vasavada AN, Danaraj J, Siegmund GP. Head and neck anthropometry, vertebral geometry and neck strength in height-matched men and women. *J Biomech*. 2008;41:114–121.
110. Vaziri S, Lockney DT, Dru AB, Polifka AJ, Fox WC, Hoh DJ. Does ossification of the posterior longitudinal ligament progress after fusion? *Neurospine*. 2019;16(3):483–491. doi: 10.14245/ns.1938286.143
111. Viano DC, Parenteau CS. Serious injury in very-low and very-high speed rear impacts. SAE Technical Paper No. 2008-01-1485.
112. Westerveld LA. Diffuse idiopathic skeletal hyperostosis (DISH): the impact of spinal ankylosis on trauma patients. Doctoral Dissertation, Utrecht University, 2011.
113. Xu Y, Wu Q. Decreasing trend of bone mineral density in US multiethnic population: Analysis of continuous NHANES 2005–2014. *Osteoporos Int*. 2018;29(11):2437-46.
114. Yanage T, Kato H, Yamamura Y, et al. Ossification of spinal ligaments. *Rinsho Sonker*. 1972;12:571–577.
